# Identification of 90 NAFLD GWAS loci and establishment of NAFLD PRS and causal role of NAFLD in coronary artery disease

**DOI:** 10.1016/j.xhgg.2021.100056

**Published:** 2021-08-24

**Authors:** Zong Miao, Kristina M. Garske, David Z. Pan, Amogha Koka, Dorota Kaminska, Ville Männistö, Janet S. Sinsheimer, Jussi Pihlajamäki, Päivi Pajukanta

**Affiliations:** 1 Department of Human Genetics, David Geffen School of Medicine at UCLA, Los Angeles, CA, USA; 2 Bioinformatics Interdepartmental Program, UCLA, Los Angeles, CA, USA; 3 Institute of Public Health and Clinical Nutrition UEF, Kuopio, Finland; 4 Turku PET Centre, Turku University Hospital, Turku, Finland; 5 Department of Medicine, UEF and Kuopio University Hospital, Kuopio, Finland; 6 Department of Experimental Vascular Medicine, Amsterdam UMC, Location AMC at University of Amsterdam, Amsterdam, the Netherlands; 7 Department of Computational Medicine, UCLA, Los Angeles, CA, USA; 8 Department of Medicine, Endocrinology, and Clinical Nutrition, Kuopio University Hospital, Kuopio, Finland; 9 Institute for Precision Health, David Geffen School of Medicine at UCLA, Los Angeles, CA, USA

**Keywords:** NAFLD, UK Biobank, GWAS, polygenic risk score, Mendelian randomization

## Abstract

The prevalence of non-alcoholic fatty liver disease (NAFLD), now also known as metabolic dysfunction-associated fatty liver disease (MAFLD), is rapidly increasing worldwide due to the ongoing obesity epidemic. However, currently the NALFD diagnosis requires non-readily available imaging technologies or liver biopsy, which has drastically limited the sample sizes of NAFLD studies and hampered the discovery of its genetic component. Here we utilized the large UK Biobank (UKB) to accurately estimate the NAFLD status in UKB based on common serum traits and anthropometric measures. Scoring all individuals in UKB for NAFLD risk resulted in 28,396 NAFLD cases and 108,652 healthy individuals at a >90% confidence level. Using this imputed NAFLD status to perform the largest NAFLD genome-wide association study (GWAS) to date, we identified 94 independent (R^2^ < 0.2) NAFLD GWAS loci, of which 90 have not been identified before; built a polygenic risk score (PRS) model to predict the genetic risk of NAFLD; and used the GWAS variants of imputed NAFLD for a tissue-aware Mendelian randomization analysis that discovered a significant causal effect of NAFLD on coronary artery disease (CAD). In summary, we accurately estimated the NAFLD status in UKB using common serum traits and anthropometric measures, which empowered us to identify 90 GWAS NAFLD loci, build NAFLD PRS, and discover a significant causal effect of NAFLD on CAD.

## Teaser

Non-alcoholic fatty liver disease (NAFLD) is underdiagnosed due to limitations in diagnostic techniques. We estimated NAFLD in the UK Biobank based on serum and anthropometric measures, which helped identify 90 NAFLD GWAS loci, build NAFLD polygenic risk scores, and discover a causal effect of NAFLD on coronary artery disease.

## Introduction

It is estimated that over 25% of adults worldwide have non-alcoholic fatty liver disease (NAFLD [MIM: 613282]), now also known as metabolic dysfunction-associated fatty liver disease (MAFLD),^1^ and an increase in NAFLD prevalence has paralleled that of other cardiometabolic disorders, such as obesity and type 2 diabetes (T2D [MIM: 125853]). The degree of steatosis (fat in the liver) can be measured through different imaging techniques, mainly using abdominal magnetic resonance imaging (MRI) and magnetic resonance spectroscopy (MRS).^2^ However, unlike anthropometric measures, such as body mass index (BMI), or biochemical measures, such as serum liver enzymes and lipids levels, abdominal MRI/MRS is not typically conducted on asymptomatic individuals, and thus NAFLD may go undiagnosed for years. Therefore, NAFLD is likely under-diagnosed due to the relative difficulty in obtaining reliable measures of liver characteristics. Moreover, NAFLD may progress to non-alcoholic steatohepatitis (NASH) and cirrhosis.^3^ However, abdominal MRI/MRS cannot identify inflammation, ballooning, or early stages of fibrosis reliably, and these can only be diagnosed through histological assessment of liver biopsy.

Due to the scarcity of abdominal MRI and liver biopsy data, NAFLD genome-wide association studies (GWASs) have remained small,^4^, ^5^, ^6^, ^7^, ^8^, ^9^, ^10^, ^11^, ^12^, ^13^, ^14^, ^15^, ^16^ the largest ones comprising ∼7,500 individuals in an MRI-based steatosis GWAS^5^ and 1,500 cases in a biopsy-based NAFLD GWAS.^15^ Thus, identifying risk loci for NAFLD has been slower than with other cardio-metabolic diseases, such as obesity, T2D, or hypercholesterolemia. Given that the diagnosis of NAFLD or NASH by either imaging or liver histology is not readily available, one alternative method for identifying individuals with likely NAFLD for GWASs is to establish a NAFLD risk score from the correlated clinical traits, such as serum liver enzymes, glucose, and lipid levels. Previously, Bedogni et al.^17^ reported the widely used fatty liver index (FLI); however, in a validation study FLI did not outperform the simple waist circumference in predicting NAFLD.^18^ The existing prediction models are usually built on a limited sample size, which restricts the robustness/accuracy of the prediction model. Although machine learning (ML) methods have also been used in predicting NAFLD,^19^ they are still limited by the small sample size and suffer from a potential overfitting problem in certain small population groups. To improve the assessment of NAFLD using serum traits, we utilized the individuals with International Classification of Diseases, Ninth Revision (ICD9)- and ICD10-based NAFLD diagnoses and liver MRI data in the extensive UK Biobank (UKB) as the ground truth for the NAFLD status in our modeling. Accordingly, using the training cohort, we built an imputation model of NAFLD and estimated the NAFLD scores (NAFLDSs) in the full UKB. Utilizing the NAFLDS as the surrogate of NAFLD, we then performed a GWAS to powerfully identify a large number of variants for NAFLD and build the polygenic risk scores (PRSs) for NAFLD.

Our prediction approach that leverages shared genetics between fatty liver disease and other metabolic disorders is also in line with the current change in the nomenclature from NAFLD to MAFLD,^1^ which emphasizes the need to better subphenotype and stratify individuals by applying more precise genetic, anthropometric, and metabolic phenotyping approaches. Thus, our results take the field forward by demonstrating that in the large UK Biobank only part of the genome-wide genetic correlations are shared between the individual metabolic and anthropometric predictor traits and NAFLDS.

The leading cause of death from NAFLD is coronary artery disease (CAD), with an estimated 5%–10% of people with NAFLD dying from CAD.^20^ It is unclear whether the increased risk of CAD mortality in NAFLD individuals is due to other metabolic traits known to be linked to CAD and correlated with NAFLD (e.g., dyslipidemia, T2D, or obesity), and thus the causal direction between NAFLD and CAD has remained elusive.^21^ It is important to establish which CAD risk factors are causal, because therapeutic interventions should be targeted to these causal risk factors. Recently, the first MR analysis designed to investigate the causal relationship between NAFLD and CAD did not identify a positive causal effect.^22^ Here, to disentangle the causal relationship between NAFLD and CAD that may be confounded by pleiotropic effects from many cardiometabolic tissues, we used a tissue-aware two-sample bi-directional MR analysis, which suggests that the genetically determined risk of NAFLD is causal for CAD.

## Material and methods

This research has been conducted using the UK Biobank Resource under application number 33934. The GTEx coronary artery *cis*-expression quantitative trait loci (eQTL) results were obtained from the GTEx portal in the version of dbGaP Accession phs000424.v8.p2. The Kuopio Obesity Surgery (KOBS) cohort was recruited at the University of Eastern Finland and Kuopio University Hospital, Finland. All individuals gave written informed consent, and the study protocol was approved by the local ethics committee. We analyzed the liver RNA sequencing (RNA-seq) and genome-wide SNP data from 259 KOBS participants.^23^

### Estimating the NAFLDS in UKB

In UKB, the true NAFLD cases were first identified using the following ICD9/10 codes: 571.5, 571.8, 571.9, K74.0, K74.6, K75.8, and K76.0, as in previous large administrative data-based studies of NAFLD prevalence and incidence.^24^ We then selected the individuals who have a liver fat percent < 5%, assessed by abdominal MRI, and no ICD9/10 code-based NAFLD diagnosis as the true healthy control individuals. We also excluded individuals with liver disease other than NAFLD from all GWAS, PRS, and MR analyses (see the GWAS analysis section below). Then to estimate the NAFLD status in the full UKB, we used the elastic net regularization to identify key predictors for the NAFLD status among the biomarker and anthropometric measurements available in UKB. Since elastic net tends to shrink the coefficients toward null, which can bias the results, we chose to perform a multivariate logistic regression to estimate the actual effect sizes of the predictors. We also compared the NAFLD scoring results obtained using an elastic net regression (penalized by weights of the predictors) with the scores obtained using a multivariate logistic regression (not penalized) and observed a high correlation in the UKB (correlation coefficient > 0.99). Table 1 shows the effect sizes for covariates that have non-zero effect sizes, estimated by the elastic net regression in our NAFLDS model.

**Table 1:** Effect sizes (betas) estimated in the NAFLDS and NAFLDS_simple models

	**NAFLDS**	**NAFLDS_simple**
GGT	0.0138	0.0144
BMI	0.0395	0.0479
Waist	0.0606	0.0714
ALT	0.0089	0.0125
AST	0.0373	0.0346
HbA1c	0.0360	NA
AST/ALT	−0.1299	−0.1794
TG	0.3499	NA
Cholesterol	−0.2850	NA
Albumin	−0.0035	NA
Age	−0.1470	−0.1722
Age^2^	0.0015	0.0018
Sex	−1.0252	−0.9153
T2D	0.4123	NA

The predictors are ranked by their importance in the random forest estimation model. NA indicates not applicable.

To evaluate the accuracy of this model, we performed a 100-fold cross-validation in UKB. In more detail, we randomly split the individuals into 100 groups, so that the training groups contained 99% of the individuals and the remaining independent 1% of the individuals were estimated based on the trained model. To estimate the importance of different predictors, we also trained a random forest model using the same predictors that we used in the NAFLDS model. The random forest was employed using the “randomForest”^25^ R package with default parameters. Next, we compared NAFLDS, FLI,^17^ hepatic steatosis index (HSI),^26^ and gamma-glutamyl transpeptidase (GGT) in estimating the NAFLD status using a receiver operating characteristic (ROC) curve. To impute the final NAFLDS in the full UKB cohort, we trained the NAFLDS model using all the individuals who have ground truth values (combining both the training and testing group) and then applied the model to the full UKB cohort. Finally, the estimated NAFLDS status was used as the surrogate for the NAFLD status in our following GWAS, PRS, and MR analyses.

### GWAS analysis

We used a linear mixed model implemented by BOLT-LMM to identify the associations between the genetic variants and selected traits (NAFLD, CAD, and the predictors of NAFLDS) while taking into account the population structure in UKB. The CAD individuals were identified using the ICD9/10, as described by Khera et al.^27^ The imputed NAFLD status was defined by the NAFLDS using the cutoff points of −1.5/1.5 (see Results for justification of these cutoff points). In total, 28,396 NAFLD cases and 108,652 healthy controls were identified at a >90% confidence level. We also included age, age^2^, sex, BMI, top 20 genotype PCs, array type, and center ID as covariates. To decrease genetic heterogeneity and avoid confounding due to multiple ethnicities and population substructures, only unrelated participants of European ancestry were included in the analysis. We also excluded individuals with liver disease other than NAFLD from the GWAS, PRS, and MR analyses using ICD9/ICD10 codes: 571.1–4, 571.6, 572.0, 572.8, 573.3, 573.8–9, K70.0–4, K70.9, K71.0–2, K71.5–9, K72.0–1, K72.9, K73.0–2, K73.8–9, K74.1–5, K75.0, K75.2–4, K75.9, K76.1–3, K76.6–9, and K77.0. To fulfill a two-sample MR requirement, we performed the CAD GWAS among the individuals who do not have a solid estimation of NAFLD status (n = 127,635).

### *cis*-eQTL analysis in the KOBS liver RNA-seq data

To identify the *cis*-eQTLs in the KOBS liver RNA-seq cohort, we first estimated the gene expression using Kallisto. Only the genes that had an estimated TPM > 0.1 in more than 90% of the KOBS liver samples were retained for the analyses. We also performed a 2-pass alignment using STAR and estimated the following technical factors: mitochondrial reads percent, mRNA reads percent, uniquely mapped rate, 5′ bias, and 3′ bias. Then we adjusted the gene expression for the technical factors, RIN, first 3 genotype PCs, and 20 SVAs. The *cis* region was defined as 1 million bases up/downstream of the transcription start site of the target genes. Using a permutation-based multiple test correction employed by fastQTL, we identified 260,748 significant *cis*-eQTL SNP-target gene pairs passing false discovery rate (FDR) < 0.05.

### Mendelian randomization analysis

Using the summary statistics that we obtained from our GWAS analysis, we explored the causal relationship between NAFLD and CAD (diagrammatically NAFLD↔CAD). We first overlapped the KOBS/GTEx liver *cis*-eQTLs and the GTEx coronary artery *cis*-eQTLs and filtered out the shared SNPs that might affect both the liver and coronary arteries. The *cis*-eQTLs that only exist in one of these tissues were identified as the tissue-aware *cis*-eQTLs. When using the imputed NAFLD status as the exposure variable, we identified the variants that are significant both in the NAFLD GWAS and liver-aware cis-eQTL analysis in the KOBS or GTEx cohort. The identified SNPs most likely affect the liver health status, reflected by the imputed NAFLD status. Then we linkage disequilibrium (LD) pruned (R^2^ = 0.2) the overlapping SNPs and treated the non-redundant SNPs as instrumental variables (IVs). When testing the causal effect of CAD on NAFLD, we included both the UKB CAD GWAS SNPs and the CARDIoGRAMplusC4D CAD GWAS SNPs^28^ as the candidate IVs and overlapped these GWAS SNPs with GTEx coronary artery-aware *cis*-eQTLs. We LD pruned (R^2^ = 0.2) the CAD GWAS *cis*-eQTLs and treated the independent CAD GWAS SNPs as IVs. Next, we used MR-PRESSO^29^ to correct for the potential horizontal pleiotropy and tested for the causal effects between the imputed NAFLD status and CAD in both directions. We also employed a heterogeneity test (Cochran’s Q test) to search for potential horizontal pleiotropy. When we used alanine aminotransferase (ALT) as a surrogate for liver health, we randomly separated UKB into 2 independent groups and performed a two-sample MR, similarly as described for NAFLD↔CAD. Since the heterogeneity test for ALT→CAD showed a sign of potential horizontal pleiotropy, we further verified the two-sample MR using the MR-egger^30^ that verified our one-way causal effect of ALT→CAD without detecting any signs of horizontal pleiotropy (see Results).

## Results

### NAFLDS model accurately assesses the NAFLD status in UKB

To impute the NAFLD status using available traits in UKB, we first identified the NAFLD and healthy control individuals using the same ICD9/10 codes (see Material and methods) for NAFLD as employed in several previous large administrative data-based studies of NAFLD prevalence and incidence.^24^^,^^31^^,^^32^ We used MRI (liver fat percent < 5%) and ICD9/10 data (no ICD9/10 NAFLD diagnosis) to identify healthy control individuals. We also excluded individuals with liver disease other than NAFLD from all GWAS, PRS, and MR analyses (see Material and methods). A total of 2,181 true NAFLD cases and 2,444 true healthy control subjects were identified. We then selected 14 NAFLD-related traits, including age, BMI, liver enzymes, blood glucose, and lipid traits as predictors and first used elastic net^33^ to select the informative traits for our NAFLDS model (see Material and methods for the detailed description of our model). All 14 predictors were kept in the elastic net model, which indicates their important role in estimating the NAFLD status. Since an elastic net model is known to shrink the coefficients toward null and thus bias results, we performed a multivariate logistic regression to train and predict the NAFLD status (see Material and methods).

**Figure 1:**
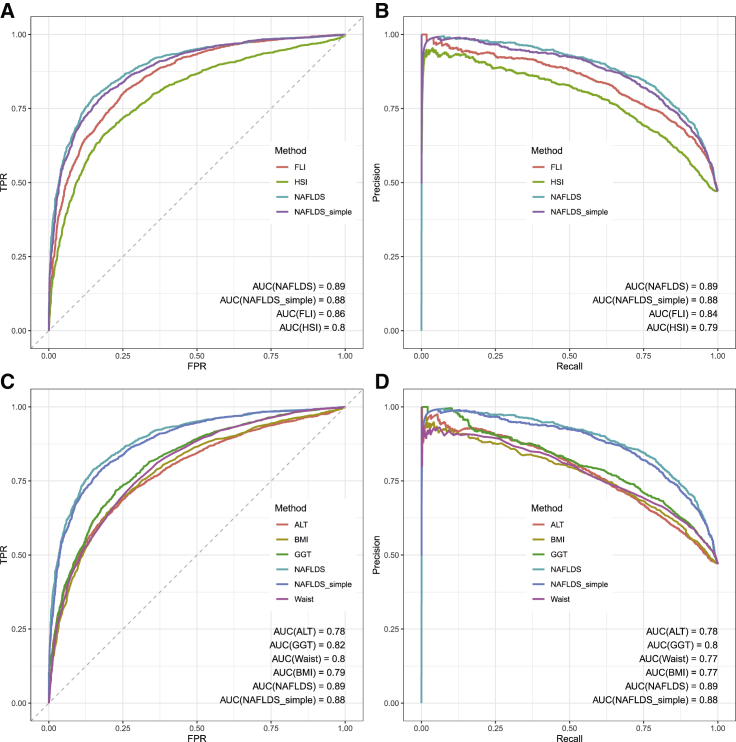
ROC and PRC plots show that NAFLDS outperformed the existing NAFLD predictors (A) As demonstrated by an ROC curve, NAFLDS outperformed FLI and HSI by achieving higher AUCs. (B) As demonstrated by a PRC plot, NAFLDS and NAFLDS_simple outperformed FLI and HIS and achieved higher AUCs. (C) In the ROC plot, NAFLDS outperforms the key predictors, ALT, GGT, BMI, and waist circumference. (D) In the RPC plot, NAFLDS outperforms the key predictors, ALT, GGT, BMI, and waist circumference.

Using a 100-fold cross-validation, we assessed the performance of NAFLDS, FLI, and HSI on predicting the NAFLD status. Figures 1A and 1B show that NAFLDS outperformed FLI and HSI in estimating the NAFLD status in the 100-fold cross-validation as well as achieved the highest area under the curve (AUC) in an ROC curve (AUC = 0.89, 95% CI = 0.88–0.90) and in a precision recall curve (PRC) (AUC = 0.89). Moreover, NAFLDS outperformed all predictor traits, including ALT, GGT, BMI, and waist, in predicting the NAFLD status. Figures 1C and 1D show the comparison between the key predictor traits and NAFLDS. We also randomly selected 80% of the samples as the training set and tested the NAFLDS model on the remaining 20% of the samples. The training/testing set shows a similar performance as the 100-fold cross-validation (Figure S1).

Moreover, we calculated the positive predictive value (PPV) and negative predictive value (NPV) of different cutoff points and set −1.5/1.5 as the low/high cutoff points of NAFLDS. These cutoff points were selected to call NAFLD cases and controls at a >90% confidence level, as is evidenced by the fact that when we applied the high cutoff point (1.5), we identified 1,188 NAFLD cases, of which 93% (1,104) were true NAFLD cases, and when we applied the low cutoff point of −1.5, we identified 1,406 NAFLD-free individuals, of whom 92% (1,287) were correctly categorized. Thus, utilizing both the high 1.5 and low −1.5 cutoff points, our NAFLDS model diagnosed the binary NAFLD status of 2,391 individuals (52% of the overall study sample) at a high accuracy (≥92% in both NAFLD cases and healthy control individuals).

To investigate the relative importance of the different predictors, we also applied a random forest model to the same training/testing groups and observed that GGT, waist circumference, and BMI ranked high as the most important predictors (Table 1). The diabetic traits, such as hemoglobin A1c (HbA1c) and T2D, were less important predictors. Thus, we trained another linear model that only relies on the liver enzymes and anthropometric measures (i.e., ALT, AST, GGT, AST/ALT, waist circumference, sex, age, age^2^, and BMI). This simplified model (NAFLDS_simple) also outperformed FLI, his, and any predictor alone in the 100-fold cross-validation (Figure 1; Figure S1). Thus, when all predictors in the NAFLDS model are not available, the NAFLDS_simple can be employed to obtain a similar performance on estimating the NAFLD status as NAFLDS has. Table 1 shows the estimated betas of both NAFLDS and NAFLDS_simple.

### The imputed NAFLD status increases power in NAFLD GWAS analysis

Since our NAFLDS model was shown to accurately predict NAFLD in the 100-fold cross-validation, we next trained the model using all the 4,625 individuals who have the ground truth NAFLD status and imputed the NAFLD status using NAFLDS in the full UKB. Using the same 1.5/−1.5 cutoff point, we observed 28,396 NAFLD cases (NAFLDS > 1.5) and 108,652 healthy control subjects (NAFLDS < −1.5) in UKB. We then performed a GWAS analysis on the two traits (i.e., the NAFLD status [n = 5,059] and the imputed NAFLD status based on NAFLDS [n = 136,804 after excluding individuals with other known liver diseases in UKB]).

In the small GWAS analysis of NAFLD status, we identified 2 NAFLD GWAS loci with 68 genome-wide significant (p value < 5E−8) variants in 2 independent LD blocks (R^2^ < 0.2). Comparing to the previously identified suggestive or significant NAFLD GWAS loci,^4^, ^5^, ^6^, ^7^, ^8^, ^9^, ^10^, ^11^, ^12^, ^13^, ^14^, ^15^ our SNPs, rs73004951 and rs2294915, replicated the previous NAFLD GWAS loci, *TM6SF2* and *PNPLA3* (Table S1). Table S2 shows the detailed summary statistics of all significant GWAS loci of the NAFLD status. Noteworthy, all of these NAFLD GWAS variants were also replicated in the GWAS analyses of the imputed NAFLD status (see below).

Given the larger sample size for the imputed NAFLD status (n = 28,396 cases with NAFLDS > 1.5 and n = 108,652 controls with NAFLDS < −1.5) when compared with the NAFLD status (n = 4,625, verified by ICD codes and MRI data), we identified substantially more significant GWAS variants for the imputed NAFLD status than in the NAFLD status GWAS analysis (see above). All in all, we identified 94 NAFLD GWAS loci for the imputed NAFLD status, with 5,187 significant (p < 5E-8) variants in 94 independent LD blocks (R^2^ cutoff, 0.2), which is 13 times more loci than reported in the previous NAFLD GWASs together (Tables S1 and S3). Figure S2 shows the QQ-plots of the p values calculated in the NAFLD and imputed NAFLD GWAS analysis. No genome-wide inflation was observed in Figure S2A (genomic inflation factor lambda = 1.00). Although Figure S2B showed a sign of higher p values than expected (lambda = 1.20), it might be caused by a true polygenic signal captured by the large sample size rather than the inflation caused by population substructure, similarly as shown in a previous paper.^34^ We further tested this hypothesis by subsampling the imputed NAFLD cohort to a similar same size (n = 6,425) as the NAFLD status. There is no sign of inflation in the subsampled GWAS analysis (Figure S2C, lambda = 1.00). Overall, we identified 90 GWAS loci that have not been identified for NAFLD before.

To assess the performance of the imputed NAFLDS, we also compared our NAFLDS GWAS loci to the previously reported NAFLD GWAS loci^4^, ^5^, ^6^, ^7^, ^8^, ^9^, ^10^, ^11^, ^12^, ^13^, ^14^, ^15^ observed using substantially smaller numbers of NAFLD cases and control subjects (see Table S1). Our imputed NAFLD GWAS replicated all of the suggestive and significant NAFLD GWAS loci that were earlier reported by several of the previous small NAFLD GWASs, including the key NAFLD loci TM6SF2 (MIM: 606563), SAMM50 (MIM: 612058), and PNPLA3 (MIM: 609567) (Table 2), while we did not replicate some of the previous NAFLD GWAS loci reported by only one of the previous small NAFLD GWASs (Table 2; Tables S1 and S3).

**Table 2:** Seven of the Previously identified NAFLD GWAS loci were observed in our imputed NAFLD status (n = 136,840) GWAS analyses at the genome-wide significant level (p < 5E−8) or subgenome-wide significant level (p < 5E−5)

**CHR**	**Gene/loci**^a^	**SNP ID**	**Beta**	**p**	**Significance level**
**Previous significant loci**					

1	MARC1 (MIM: 614126)	rs2642438	−1.09E−3	0.28	–
2	GCKR (MIM: 600842)	rs1260326	9.08E−3	2.00E−22	genome
2	GCKR	rs780094	8.47E−3	1.70E−19	genome
4	HSD17B13 (MIM: 612127)	rs9992651	5.23E−3	5.40E−07	subgenome
7	–	rs343062	−8.17E−4	3.80E−01	–
8	PPP1R3B (MIM: 610541)	rs4240624	3.90E−3	1.30E−02	nominal
16	ZFP90-CDH1 (MIM: 609451)	rs698718	−1.70E−3	1.10E−01	–
19	NCAN (MIM: 600826)	rs2228603	−8.47E−3	8.60E−07	subgenome
19	TM6SF2 (MIM: 606563)	rs58542926	−9.55E−3	2.70E−08	genome
22	SAMM50 (MIM: 612058)	rs3761472	−8.97E−3	9.80E−13	genome
22	SAMM50	rs2143571	−5.73E−3	1.40E−06	subgenome
22	PNPLA3 (MIM: 609567)	rs738409	−1.19E−2	3.60E−27	genome
22	IL17RA (MIM: 605461)	rs5748926	1.13E−05	9.90E−01	–
22	PARVB (MIM: 608121)	rs5764455	−2.33E−04	8.00E−01	–

**Previous suggestive loci**					

1	LYPLAL1 (MIM: 616548)	rs12137855	1.85E−3	9.80E−02	–
2	FABP1 (MIM: 134650)	rs72943235	−7.26E−4	8.70E−01	–
8	TRIB1 (MIM: 609461)	rs2980888	1.00E−2	7.30E−24	genome
8	TRIB1	rs2954021	1.06E−2	3.50E−31	genome
8	FDFT1 (MIM: 184420)	rs2645424	1.53E−4	8.70E−01	–
19	MBOAT7 (MIM: 606048)^b^	rs641738	9.22E−4	2.6E−03	nominal

**Previous unidentified loci**					

17	GRB2 (MIM: 108355)	rs5015881	−7.99E−3	1.60E−08	genome

Loci were previously identified in NAFLD GWASs.^4^, ^5^, ^6^, ^7^, ^8^, ^9^, ^10^, ^11^, ^12^, ^13^, ^14^, ^15^, ^16^

a The Gene/loci column shows the nearest gene of the identified NAFLD variant.

b This variant was derived from a previous study^16^ that performed a meta-analysis of rs641738 instead of a full GWAS.

Next, we overlapped the GWAS loci obtained using the imputed NAFLD status with liver *cis-*eQTLs identified from 208 liver RNA-seq samples from GTEx and 259 liver RNA-seq samples from KOBS. In total, the GWAS variants regulated 50 liver eQTL target genes (eGenes) in either GTEx or KOBS as *cis*-eQTLs (FDR < 0.05). Among the 50 liver eGenes, 19 were observed in both GTEx and KOBS. These results provide potential targets for co-localization analyses in the future fine mapping studies to further confirm the co-location between the GWAS and *cis*-eQTL variants at each of these loci. Table S4 lists the SNP-liver eGene pairs at the imputed NAFLD GWAS loci. Noteworthy, 7 of the 19 eGenes are Human Leukocyte Antigen (*HLA*) genes that replicated the recent report by Yoshida et al.^35^

### The imputed NAFLD (NAFLDS) GWAS loci overlap largely with liver enzyme (ALT and GGT) GWAS loci

Since the NAFLDS is predicted using 14 different predictors, we further investigated whether the imputed NAFLD status (n = 28,396 cases with NAFLDS > 1.5 and n = 108,652 control subjects with NAFLDS < −1.5) helps to identify GWAS loci that cannot be identified by the individual predictors of the NALFDS model. We chose five predictors to represent the five categories of predictors (triglycerides for serum lipid, HbA1c for serum glucose, ALT and GGT for liver enzyme, and BMI for overall body obesity) and then performed GWASs for these 5 representative predictors and compared their GWAS variants with the significant imputed NAFLD GWAS variants. Table 3 shows that the majority of the significant imputed NAFLD GWAS variants were replicated in the GWAS of the 5 predictors. Importantly, among the tested predictors, the liver enzyme GWAS loci overlapped largely with the imputed NAFLD (NAFLDS) GWAS. Specifically, we observed an overlap of 64.77% between ALT and NAFLDS GWAS loci and an overlap of 72.49% between the GGT and NAFLDS GWAS loci, while the overlaps for HbA1c (32.18%) and BMI (6.48%) were much smaller (Table 3). Although this fits the assumption that liver enzymes directly represent the liver health status, it also suggests, however, that only part of the genome-wide genetic correlations are shared between the individual predictor traits and NAFLDS, as not all liver enzyme, TG, glucose, and BMI GWAS loci confer the genetic risk of NALFD. Thus, the NAFLDS model may help capture the critical combination of the composite trait GWAS loci that confer the genetic NAFLD risk.

**Table 3:** The number of GWAS variants shared by the imputed NAFLD status and predictors

	**Triglycerides**	**HbA1c**	**BMI**	**ALT**	**GGT**	**All**
Shared SNPs	3,366	1,669	336	3,360	3,760	4,890
Percentage	64.89%	32.18%	6.48%	64.77%	72.49%	94.27%

Hba1c, hemoglobin A1c; BMI, body mass index; ALT, alanine aminotransferase; GGT, gamma-glutamyl transpeptidase.

When comparing the imputed NAFLD GWAS variants with the GWAS variants of the predictors, 110 GWAS variants of imputed NAFLD are specific to the imputed NAFLD and do not overlap or are not in LD (R^2^ < 0.8) with the significant GWAS variants of these predictors. We then tested the associations between the 110 GWAS variants with all 14 predictors and identified a set of 8 variants in tight LD (R^2^ > 0.80) in a 33-kb region on chromosome 17 that are not associated with any predictors (all predictor traits with p > 5E-8). These 8 imputed NAFLD GWAS variants belong to the same LD block overlapping the Growth Factor Receptor Bound Protein 2 (*GRB2*) gene, suggesting this gene as a possible underlying gene, though further fine mapping and functional studies are warranted to identify the actual regional NAFLD gene. Table S5 shows the summary statistics of the 8 NAFLD GWAS SNPs at this LD block in the *GRB2* region that do not overlap with the GWAS loci detected by any predictors (all predictor traits with p > 5E-8). Noteworthy, as there are other regional variants that do not reside in this LD block that are associated with waist-hip ratio adjusted BMI and waist-hip ratio in previous GWASs,^36^ the overall *GRB2* region thus shows genetic effects on multiple metabolic traits beyond the imputed NAFLDS.

### A PRS model of the imputed NAFLD status predicts the risk of NAFLD in UKB

**Figure 2:**
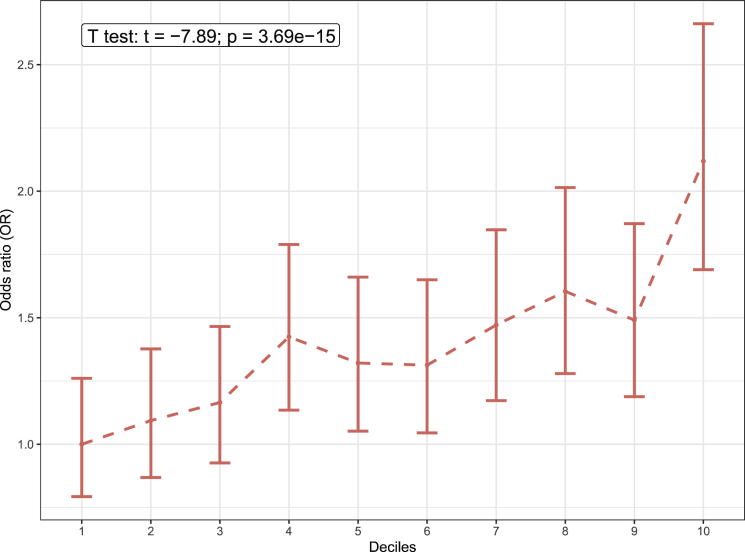
The ORs of NAFLD for the decile compared to the people with the lowest 10% NAFLD PRS score The error bar shows the 95% confidence interval of the estimated OR. The x axis shows the 10 deciles divided by the NAFLD PRS score. The annotation box indicates the result comparing the inverse normal transformed PRS scores between the NAFLD cases and control subjects using a Student’s t test.

To investigate how the imputed NAFLD GWAS variants predict individual risk of NAFLD, we constructed a PRS model for the imputed NAFLD status in UKB. To train and build the NAFLD PRS model, we separated UKB into 3 independent groups: training set (n = 99,823), test set (n = 34,833), and validation set (n = 5,059). The validation set contained all the case/control individuals whose NAFLD status was verified by ICD codes or MRI scan while training, and the test set contained the individuals whose NAFLD status were imputed by the NAFLDS model. We first performed a GWAS analysis of the imputed NAFLD status in the training set to establish the effect sizes (beta) of all variants on the NAFLD risk. Then we investigated the effectiveness of the PRS model in the test set using different combinations of LD pruning thresholds (R^2^ from 0.2 to 0.8) and p value thresholds (p from 5 × 10^−8^ to 0.1) using the polygenic score function in plink.^37^ In the test set, we compared the PRS score of the NAFLD cases and healthy control individuals. Then we divided the individuals into 10 deciles based on their PRS scores and assessed the odds ratio (OR) of having NAFLD in each of the 10 deciles when compared to the lowest decile. Figure S3 shows that in the test set, the imputed NAFLD cases always have a higher PRS value compared to the imputed healthy controls, and the top decile that has the highest PRS shows an OR between 1.7 and 2.4. We picked the combination of R^2^ < 0.8 and p < 0.1 as the best thresholds, because this provided the most significant difference in the PRS between the imputed NAFLD cases and the imputed controls and also identified the highest OR between the 10^th^ decile versus the 1^st^ decile. Finally, we applied this model to the validation set and observed a concordant difference of the PRS between the NAFLD cases (identified by ICD codes) and control subjects (verified by MRI data). Figure 2 shows that the NAFLD cases have a significantly higher PRS compared to the control subjects (t = −7.89, p = 3.69 × 10^−15^), while the OR of the 10^th^ decile when compared to the 1^st^ decile is 2.1.

### NAFLD exhibits a causal effect on CAD

To determine whether there is a causal relationship between NAFLD and CAD risk, we performed a two-sample bi-directional MR analysis using the imputed NAFLD status as the surrogate of the ground truth NAFLD risk in UKB. MR requires the use of proper IVs, which are often SNPs that are known to significantly contribute to the exposure (GWAS SNPs). In UKB, we treated the 5,187 significant GWAS variants of the imputed NAFLD status as the candidate IVs. To perform GWAS in two independent cohorts required by a two-sample MR setup, we also performed a GWAS analysis of CAD among the individuals who do not have a predicted NAFLD status (n = 127,635). With only 17,188 CAD cases in UKB, we identified fewer significant CAD GWAS SNPs (n = 841 without LD pruning) than in the imputed NAFLD GWAS (n = 5,187 without LD pruning). Therefore, we also included the reported known CAD GWAS SNPs from the large Cardiogram meta-study^28^ into our analysis to expand our CAD GWAS SNP pool.

Moreover, the IVs used in an MR analysis should preferably have a known function to decrease horizontal pleiotropy, as pleiotropy can lead to misleading MR results.^29^ To refine the NAFLD and CAD GWAS SNPs to those with a plausible function in the liver and coronary arteries, respectively, we determined which of the NAFLDS and CAD GWAS SNPs are *cis-*eQTLs in their respective tissues. We used RNA-seq data of 259 liver biopsies from KOBS to identify the liver *cis-*eQTLs. We also downloaded the *cis-*eQTLs identified in the liver and coronary artery tissue from GTEx v.8 and excluded *cis-*eQTL SNPs that overlapped between the liver and coronary arteries to avoid including as IVs these SNPs that function as *cis*-eQTLs in both tissues. In total, 58,147 shared *cis*-eQTLs were identified in both KOBS and GTEx liver cohorts, and 464,236 *cis*-eQTLs were identified in the GTEx coronary artery samples. We then obtained our final list of candidate IVs for NAFLDS and CAD by overlapping the respective *cis*-eQTLs with the significant (p < 5E-8) NAFLDS or CAD GWAS SNPs.

**Figure 3:**
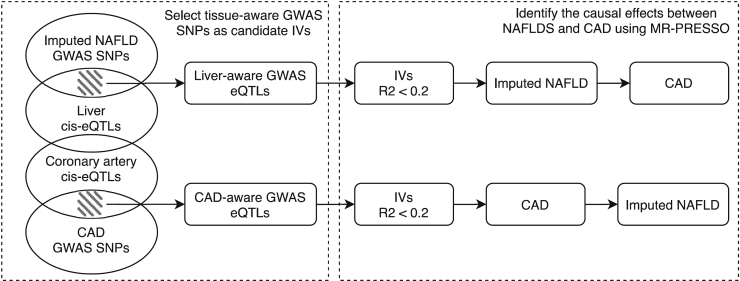
Workflow of combining liver/coronary artery *cis*-eQTL and UKB GWAS variants to a tissue-aware, bi-directional MR between imputed NAFLD and CAD

Figure 3 shows the framework of our MR models. Using our approach described above to obtain the tissue-aware eQTL NAFLDS IVs, we first discovered 5 independent SNPs (R^2^ ≤ 0.2) that are associated with NAFLD status in UKB and are liver, but not coronary artery, *cis*-eQTLs (FDR < 0.05). We identified a significant positive causal effect (beta = 0.16, p value = 5.9 × 10^−3^) of NAFLDS on CAD in UKB. To reduce the potential of pleiotropy, we used MR-PRESSO,^29^ which corrects for potential horizontal pleiotropy in the MR analysis. Moreover, we employed a heterogeneity test and again did not identify any evidence of pleiotropy (Q = 1.6, p = 0.66). To test the potential reverse causal effect of CAD on NAFLDS, we identified 18 independent SNPs (R^2^ ≤ 0.2) that are both CAD GWAS SNPs and coronary artery, but not liver, *cis*-eQTLs. Using MR-PRESSO to correct for the potential horizontal pleiotropy, we did not find a significant causal effect of CAD on NAFLDS (beta = 0.28, p = 0.24).

To further verify the direction of the causal effect of NAFLD on CAD, we performed a similar two-sample MR using ALT as the surrogate of liver health. Similarly, we observed a significant causal effect of high ALT level on the risk of CAD (beta = 0.017, p = 0.014) using MR-PRESSO, while the reverse causal effect (CAD→ALT) remained insignificant (beta = 0.51, p = 0.42. Although MR-PRESSO did not identify any potential pleiotropy, the heterogeneity test showed a slight sign of pleiotropy (Q = 43, p = 2.6E-2). Therefore, we further utilized MR-egger and verified the causal effect of ALT on CAD. In more detail, MR-egger also identified the significant causal effect of ALT on CAD (beta = 0.048, p = 0.031), while no sign of pleiotropy was observed (p.pleio = 0.15). Table S6 shows the summary statistics of all IVs utilized in the MR analysis.

In summary, we identified the IVs for the MR analyses using the GWAS SNPs of imputed NAFLD and CAD in the UKB and refined these IVs to those with functional evidence in their respective tissues by selecting the tissue-aware IVs. Our tissue-aware bi-directional MR analysis demonstrated that NAFLD causally increases the risk of CAD and did not identify any evidence of reverse causality (i.e., CAD causing increased NAFLD).

## Discussion

We used the UKB cohort to develop an estimation model of NAFLD. By combining the relevant serum traits (i.e., liver enzymes, lipids [triglycerides, cholesterol], diabetes-related traits [HbA1c, T2D status], age, sex, waist circumference, and BMI), our imputed NAFLDS achieved a high accuracy on NAFLD (AUC = 0.89) and outperformed the existing FLI^17^ index, HSI^26^ index, and the key liver enzymes, ALT and GGT (Figure 1). Since the predictors are non-independent traits, the estimated betas cannot directly be used to infer the importance of the predictors in NALFD. Thus, we also employed a random forest method to predict NAFLD with these same predictors and identified that GGT, waist circumference, and BMI are the most important predictors of NAFLD in UKB.

When identifying NAFLD predictors using elastic net, we used the serum HbA1c and T2D status to represent glucose metabolism instead of serum glucose levels, because the glucose levels were not taken after overnight fasting in UKB, which may bias them. It is suggested that serum glucose and lipid levels are independent predictors for NAFLD,^17^^,^^38^ and that GGT is the only liver enzyme that is an independent predictor for NAFLD.^17^ However, using only the anthropometric measures and liver enzymes in the UKB cohort, our NAFLDS_simple model achieved a similar power in predicting NAFLD status as our NAFLDS model that also utilized the lipid and glucose traits. Thus, our NAFLDS_simple model emphasizes the importance of liver enzymes and anthropometric measures in predicting the NAFLD status.

Since the NAFLDS model showed a high accuracy in predicting NAFLD cases and healthy control subjects when employing the cutoff points of −1.5/1.5, we used it to predict 28,396 NAFLD cases and 108,652 healthy individuals at >90% confidence level in UKB. This sample size increased the power of our GWAS analysis and resulted in the discovery of 94 independent NAFLD GWAS loci (p < 5E-8, R^2^ < 0.2), which is 13 times more loci than reported previously in NAFLD GWASs^4^ (Table S1). Importantly, we observed large overlaps between the liver enzyme (ALT and GGT) GWAS loci and our NAFLDS GWAS loci, in line with the previous biobank study in the Million Veteran Program, reporting ALT as a noninvasive NAFLD proxy.^39^ However, NAFLDS did outperform both liver enzymes in our prediction model that used the ICD9/10-based NAFLD cases and MRI-based non-NAFLD control subjects as the ground truth (Figure 1), which together with the 65%–72% GWAS overlaps between the liver enzymes and NAFLDS (Table 3) indicate that the NAFLDS model still captures additional diagnostic and genetic information beyond the liver enzymes. It is also interesting that no more than 32.18% and 6.48% of the HbA1c and BMI GWAS loci overlapped with the NAFLDS GWAS loci (Table 3). Taken together, these data suggest that only part of the genome-wide genetic correlations are shared between the individual predictor traits and NAFLDS, thus further suggesting that NAFLDS captures the critical combination of the composite trait GWAS loci contributing to NAFLD, while not all liver enzyme, TG, glucose, and BMI GWAS loci confer the genetic risk of NALFD. This information on shared genetic risks and their biological overlap between NAFLD and other metabolic disorders can ultimately help develop future genotype-based precision medicine approaches through better stratification of individuals.^40^

Our NAFLDS GWAS results that uniquely share some but not all GWAS loci with the metabolic and anthropometric component traits also support the future goals of the recent nomenclature shift from NAFLD to MAFLD that aim to better subphenotype the heterogeneous group of fatty liver individuals with metabolic dysfunction.^1^ The shift from NALFD to MAFLD reflects the recognition in the field of study that more precise genetic, anthropometric, and metabolic phenotyping approaches are needed to better assess the complex MAFLD phenotype shaped by interactions of genetic predisposition with environmental factors and components of the metabolic syndrome.^1^ Recent genetic studies with the previously known key GWAS variants also support the usefulness of the MAFLD criteria compared to NAFLD criteria in identifying individuals who benefit from genetic testing.^1^^,^^41^^,^^42^

Using the effect sizes estimated from the GWAS variants, we also built and tested a PRS model that predicted the NAFLD risk in UKB. In the validation set where individuals’ NAFLD case/control status was verified by the ICD codes or MRI data, the individuals who have a higher NAFLD PRS score (top 10%) exhibited an OR of 2.1 when compared to the individuals who have the lowest (bottom 10%) NAFLD PRS score. The NAFLD individuals also show a significantly higher NAFLD PRS score compared to the healthy control subjects (p = 3.69 × 10^−15^). We recognize that the use of ICD coding may underestimate the prevalence of NAFLD, which perhaps contributes to the relatively low OR in the PRS. Nevertheless, both the GWAS and PRS analyses demonstrate that the NAFLD status imputed by our NAFLDS model greatly increased the power in identifying genetic variants associated with the risk of NAFLD for future follow-up studies.

It is difficult to distinguish the specific contribution of NAFLD on CAD from the other risk factors shared by NAFLD and CAD. For example, obesity is a known risk factor for both NAFLD and CAD. Thus, it is important to avoid the potential horizontal pleiotropy in the MR analysis when investigating the causal relationships between NAFLD and CAD. Here, we included BMI as a covariate to identify the GWAS variants that are associated with NAFLDS/CAD without being mediated by the obesity status (BMI). Furthermore, we combined the GWAS variants and tissue-aware *cis*-eQTLs to identify the GWAS SNPs that affect gene expression preferentially in the liver or coronary arteries. These tissue-aware *cis*-eQTL GWAS variants could thus possibly exhibit a direct causal tissue-specific role in the development of NAFLD/CAD. This design that takes advantage of the transcriptomics data will define IVs well and thus help mitigate a key current shortcoming of MR (i.e., inclusion of pleiotropic IVs that affect multiple phenotypes outside their effects on exposure in MR). Accordingly, using the tissue-aware *cis*-eQTL GWAS SNPs as IVs and applying MR-PRESSO should reduce the potential pleiotropy and thus improve the robustness of the MR analysis.

Although we used independent samples for training and validating NAFLDS in the UKB, the FLI and HSI models were originally trained in other cohorts than UKB.^17^^,^^26^ Thus, there is a possibility that a different population background or hidden covariates may bias our NAFLDS estimation when compared to FLI and HSI. Another limitation of our study is that our analyses are limited to individuals of European ancestry in order to avoid genetic heterogeneity. Thus, to explore external generalizability beyond UKB, future studies should extend this approach and its comparisons with FLI and HSI to additional cohorts and more diverse populations as more biobank data emerge. Other caveats are that the imputation of genetic risk for NAFLD can reflect liver damage and overall adiposity, rather than NAFLD per se, and that we cannot rule out some case/control misclassification. While we recognize these factors as limitations in any risk scoring system of NAFLD,^17^, ^18^, ^19^ the fact that we observed the previous NAFLD GWAS signals in our study (Table 2; Table S1^4^, ^5^, ^6^, ^7^, ^8^, ^9^, ^10^, ^11^, ^12^, ^13^, ^14^, ^15^) suggests that in general the imputed NAFLD scoring does detect NAFLD loci, although additional future validations are needed to further confirm that. We also consider that even though liver biopsy and MRI still remain the gold standard in diagnosing NAFLD, it is still useful to be able to quickly score the NAFLD risk based on only blood biomarkers in order to at least detect liver damage related to these common NAFLD risk factors. Metabolically driven common liver damage is highly likely related to the development of NAFLD.^43^ Finally, to address the caveat that MR cannot fully distinguish between horizontal pleiotropy and direct causal effects, we focused on tissue-aware *cis*-eQTL IV SNPs with potentially fewer pleiotropic effects, an approach not used before to address this key MR limitation.

In summary, we used key clinical metabolic measurements to build the NAFLDS model that is easy to employ and outperforms the existing NAFLD estimation model in UKB. When some serum traits, such as HbA1c, triglycerides, and cholesterol, are not available, our NAFLDS_simple model can be used to predict the NAFLD status. Using the imputed NAFLD status in UKB, we identified 94 independent NAFLD GWAS loci, of which 90 have not been identified before. Moreover, the power boost from the sample size (28,396 NAFLD cases and 108,652 healthy controls) also helped us successfully build a PRS model that shows a significant difference between the NAFLD cases and healthy controls. Furthermore, we combined the GWAS variants and tissue-aware *cis*-eQTLs to identify the GWAS SNPs that affect gene expression preferentially in the liver or coronary arteries. Using these tissue-aware *cis*-eQTL GWAS SNPs as IVs and applying MR-PRESSO to avoid the potential pleiotropy, we identified the putative one-way causal path from NAFLD to CAD. This result was further supported by the observed causal path from ALT to CAD using the same tissue-aware IV design in MR. Our non-invasive NAFLD model in the UKB cohort can next be implemented to other large biobanks to further investigate these results and advance our understanding of genetic predisposition to common metabolically driven liver damage and ultimately NAFLD.

## Data and code availability

UKB data are available for bona fide researchers through an application process (see web resources). The GTEx dataset is available from the NIH dbGAP, study number phs000424.v8.p2. The published article includes all other analysis results generated during this study.

## Web resources

Biobank, https://www.ukbiobank.ac.uk/learn-more-about-uk-biobank/contact-us
dbGaP, https://www.ncbi.nlm.nih.gov/projects/gap/cgi-bin/study.cgi?study_id=phs000424.v8.p2
OMIM, http://www.omim.org

## Supplemental information

Document S2. Tables S1–S6

Document S3. Article plus supplemental information
