# Supplementary material for: Identification of 90 NAFLD GWAS loci and establishment of NAFLD PRS and causal role of NAFLD in coronary artery disease
Source: HGG Adv. 2021 Aug 24;3(1):100056. doi: 10.1016/j.xhgg.2021.100056 (PMC8756520; doi:10.1016/j.xhgg.2021.100056)
Supplement: Document S1. Figures S1–S3 [file mmc1.pdf]

HGGA, Volume *3*

Supplemental information

Identification of 90 NAFLD GWAS loci and establishment of NAFLD PRS and causal role of NAFLD in coronary artery disease

Zong Miao, Kristina M. Garske, David Z. Pan, Amogha Koka, Dorota Kaminska, Ville Männistö, Janet S. Sinsheimer, Jussi Pihlajamäki, and Päivi Pajukanta

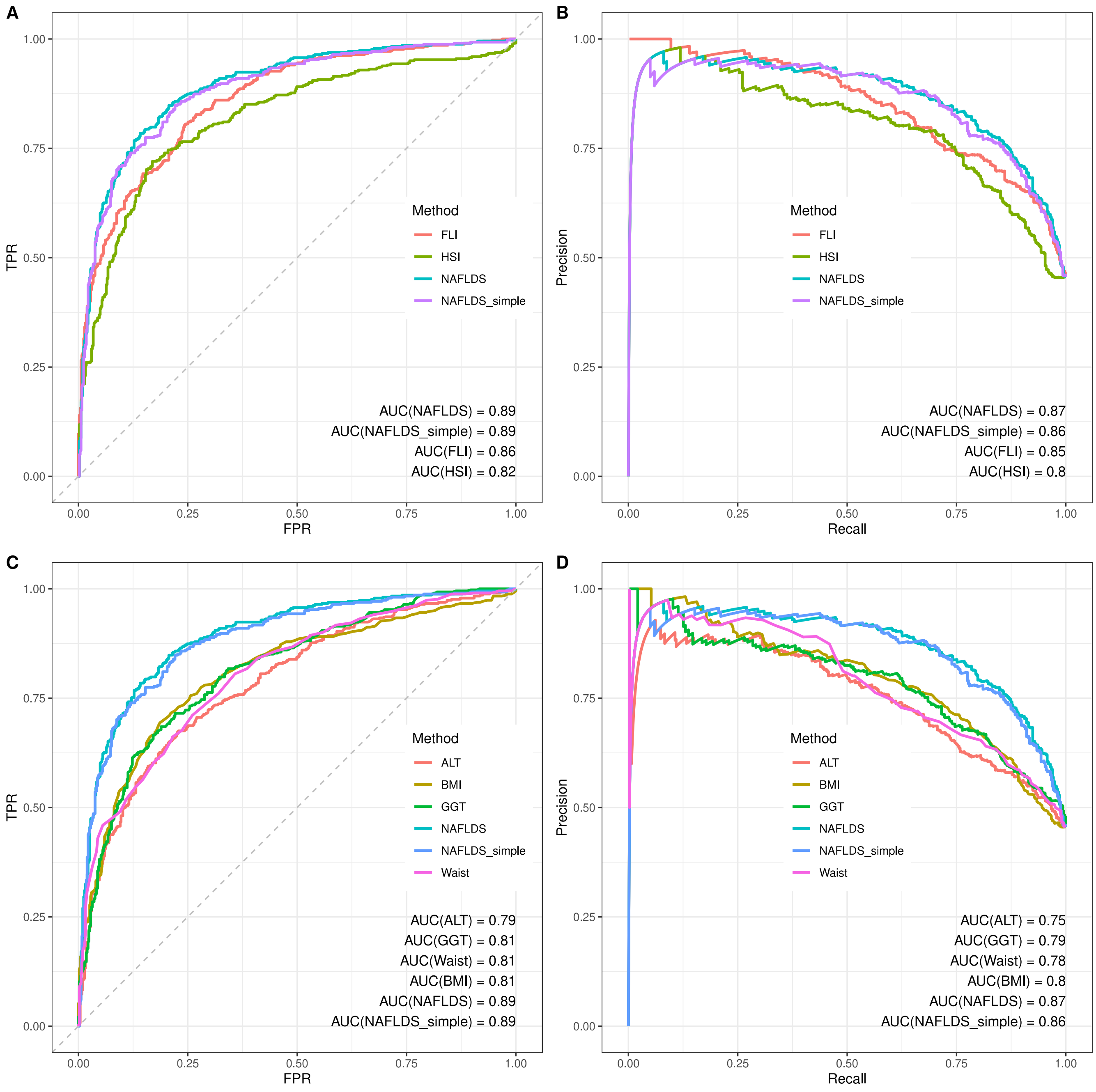

Figure S1. When we randomly separated the NAFLD cohort into 2 independent groups (80% training, 20% testing), ROC and PRC plots show that NAFLDS outperformed the existing NAFLD predictors. A). As demonstrated by a ROC curve, NAFLDS outperformed FLI and HSI by achieving higher AUCs. B) As demonstrated by a PRC plot, NAFLDS and NAFLDS_simple outperformed FLI and HIS by achieving higher AUCs. C) In the ROC plot, NAFLDS outperforms the key predictors, ALT, GGT, BMI, and waist circumference. D) In the RPC plot, NAFLDS outperforms the key predictors, ALT, GGT, BMI, and waist circumference.

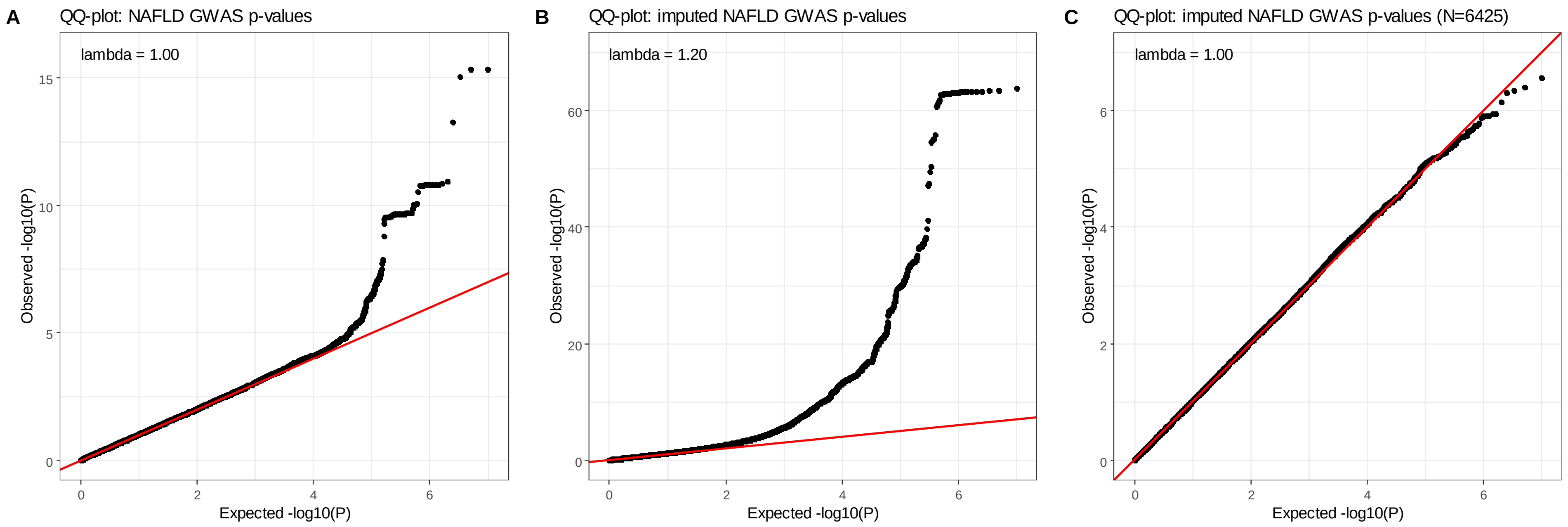

Figure S2. QQ-plot of the GWAS p-values shows no overall inflation. In each plot, the x-axis shows the expected p-values, and the y axis shows the observed p-values. The red line indicates the x=y line. Lambda indicates the genomic inflation factor. A) The QQ-plot of the p-values in NAFLD GWAS analysis. B) The QQ-plot of the p-values in imputed NAFLD GWAS analysis. C) The QQ-plot of the p-values in the subsampled (N = 6,425) imputed NAFLD GWAS analysis.

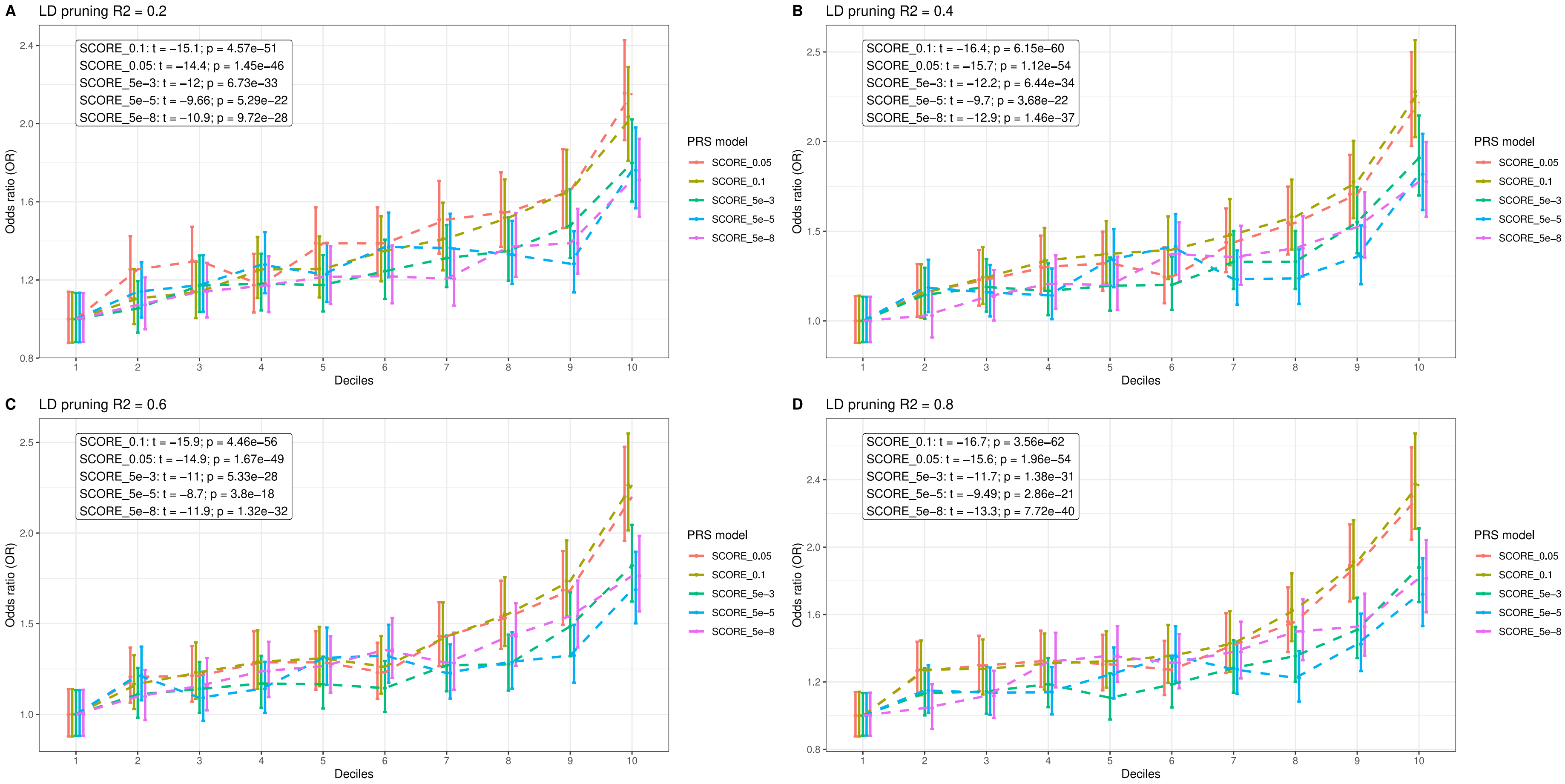

Figure S3. When building the PRS model, we tested the PRS model in the test set (n=34,833) with different combinations of R2 (0.2-0.8) and p value thresholds (5×10^{-8}-0.1). In each plot, the x axis shows the 10 deciles divided by the PRS score and y axis shows the ORs of having NAFLD when comparing to the individuals with the lowest 10% NAFLD PRS score. The error bar shows the 95% confidence interval of the estimated OR. The annotation box indicates the results when comparing the inverse normal transformed PRS scores between the NAFLD cases and controls using a student t test.
